# Is global humeral head offset related to intramedullary canal width? A computer tomography morphometric study

**DOI:** 10.1186/s40634-018-0148-2

**Published:** 2018-09-12

**Authors:** Johannes Barth, Jérôme Garret, Achilleas Boutsiadis, Etienne Sautier, Laurent Geais, Hugo Bothorel, Shoulder Friends Institute, Arnaud Godenèche

**Affiliations:** 1 Department of Orthopaedic Surgery, Centre Osteoarticulaire des Cèdres, Grenoble, France; 2 Clinique du Parc, Lyon, France; 3 Orthopaedic Surgery, University Hospital of Saint Etienne, Saint-Priest en Jarez, France; 4 Move-Up SAS, Alixan, France; 5 ReSurg SA, Chemin de la Vuarpillière 35, 1260 Nyon, Switzerland; 6 Shoulder Friends Institute, Paris, France; 7 Ramsay Générale de Santé, Hôpital Privé Jean Mermoz, Centre Orthopédique Santy, Lyon, France

**Keywords:** Intramedullary canal width, Humeral offset, Correlation, Association, Proximal humerus, Implant design, Endosteal, CT

## Abstract

**Background:**

While most anatomic TSA stems allow some intra-operative adjustments, the default configuration assumes that head offset is directly proportional to stem diameter. Some authors reported that humeral head diameter is proportional to intra-medullary canal width and humeral head offset, but none investigated the direct relationship between head offset and endosteal measurements. The purpose of the study was to determine whether global humeral head offset is proportional to intramedullary canal width at the distal metaphysis and proximal diaphysis.

**Methods:**

We analyzed 100 Computed Tomography shoulder scans of patients aged 59.1 ± 20.5 with no signs of gleno-humeral arthritis nor humeral dysplasia. The width of the intramedullary diaphyseal canal was determined at four transverse sections 65, 70, 100 and 105 mm below the head center. The inter-observer agreement was excellent for intramedullary canal width (ICC = 0.96), head diameter (ICC = 0.97) and global head offset (ICC = 0.85). Correlations were analysed using Pearson’s coefficients and multivariable regressions were performed to determine associations between head offset and five independent variables (gender, age, intramedullary canal width, head diameter).

**Results:**

Global head offset was negatively correlated with head diameter (*r* = − 0.31, *p* = 0.002), but not correlated with intramedullary canal width (*r* = − 0.11, *p* = 0.282). Multivariable regression confirmed that global head offset was independently associated with head diameter (beta = − 0.15, *p* = 0.005), but not with intramedullary canal width (beta = 0.06, *p* = 0.431).

**Conclusions:**

The present study revealed that humeral offset is not correlated with intramedullary canal width. Implant manufacturers and shoulder surgeons should be aware of the subtle morphologic features, to enhance humeral stem design and restore native anatomy.

## Background

Success of total shoulder arthroplasty (TSA) requires accurate restoration of anatomy (Godeneche et al., 2002; Irlenbusch et al., 2011; Pearl, 2005; Pearl et al., 2009; Wirth et al., 2007), as even small discrepancies between native and prosthetic geometry could trigger pain and compromise function (Kadum et al., 2016; Pearl et al., 2002; Pearl & Volk, 1996). For these reasons, most commercially-available anatomic TSA stems allow some intra-operative adjustments of humeral head offset – the distance between the head center and the diaphyseal axis – using ‘telephone dial’ or linear peg-hole configurations (Boileau & Walch, 1997; Irlenbusch et al., 2011; Pearl et al., 2009).

Numerous studies investigated the anatomy of the proximal humerus, first using 3D reconstructions of fresh or dry cadaver bones (Boileau & Walch, 1997; Hertel et al., 2002; McPherson et al., 1997; Pearl & Volk, 1996; Robertson et al., 2000; Roche et al., 2006), and more recently using X-rays (Boileau & Walch, 1997; Hertel et al., 2002; McPherson et al., 1997; Pearl & Volk, 1996; Robertson et al., 2000; Roche et al., 2006) or computed tomography (CT) scans (Aroonjarattham et al., 2009; Bockmann et al., 2016; Boileau et al., 2008; Deladerriere et al., 2012; Jia et al., 2016; Johnson et al., 2013; Kadum et al., 2016; Matsumura et al., 2014; Matsumura et al., 2016; Saka et al., 2015; Vlachopoulos et al., 2016; Zhang et al., 2016). Some authors reported that humeral head diameter is proportional to both intra- and extra-medullary humeral diameters (McPherson et al., 1997) as well as to humeral head offset (Pearl & Volk, 1996), but none investigated the direct relationship between head offset and endosteal measurements.

While some anatomic humeral components are designed with head offset proportional to stem size, others are designed with constant head offset regardless of stem size (Table 1). To the authors’ knowledge, there are no anatomic studies that investigated the correlation between native head offset and intramedullary canal width. The purpose of this study was therefore to determine the relationship between head offset and intramedullary canal width in the proximal humerus. The hypothesis was that native head offset is not correlated with intramedullary canal width.

**Table 1:** Design characteritics of humeral stems by different manufacturers

Manufacturer	Stem brand	Stem size	Distal diameter (mm)	Offset (mm)
Wright Medical	AscendFlex	1	–	6
		3	–	6.4
		5	–	7
		7	–	8.2
		9	–	10
Exactech	Equinoxe	7–100	7	7.5
		9–105	9	7.5
		11–110	11	8.5
		13–115	13	9.5
		15–120	15	9.5
		17–125	17	9.5
Zimmer	Trabecular metal	6–130	6	7.9
		8–130	8	7.9
		8–170	8	7.9
		9–130	9	7.9
		10–130	10	7.9
		10–170	10	7.9
		11–130	11	7.9
		12–130	12	7.9
		12–170	12	7.9
		13–130	13	7.9
		14–130	14	7.9
		14–170	14	7.9
		15–130	15	7.9
		16–130	16	7.9
		17–130	17	7.9
		18–130	18	7.9
DePuy Synthes	Global FX	6–120	6	7.4
		8–130	8	7.4
		10–140	10	7.4
		12–150	12	7.4
		6–160	6	7.4
		8–200	8	7.4
		10–210	10	7.4
		12–220	12	7.4

–Not applicable, short stem

## Methods

From their databases of pre-existing shoulder CT images, the authors selected 100 scans that had sufficient resolution (slice thickness 0.5 mm, with a 64 slices CT scanner) and length (> 11 cm of proximal humeri), excluding shoulders with signs of: (i) osteoarthritis or rheumatoid arthritis, (ii) Hill Sachs lesions, (iii) humeral head necrosis or deformities, and (iv) mal-unions secondary to displaced humeral neck fractures. The cohort comprised 62 men and 38 women aged 59.1 ± 20.5 years (range, 18–96). The patients presented with fractures of the scapula or clavicle (45), rotator cuff tears (39), shoulder dislocation or instability with no signs of osseous damage at the proximal humerus or glenoid (9), tumor at the scapula or distal humerus (5), calcific tendinitis (1) and thoracic syndrome (1). All patients provided informed consent to use of their images and data for research and publishing purposes. As the study was performed using pre-existing CT scans, institutional review board (IRB) approval was not required.

**Fig. 1:**
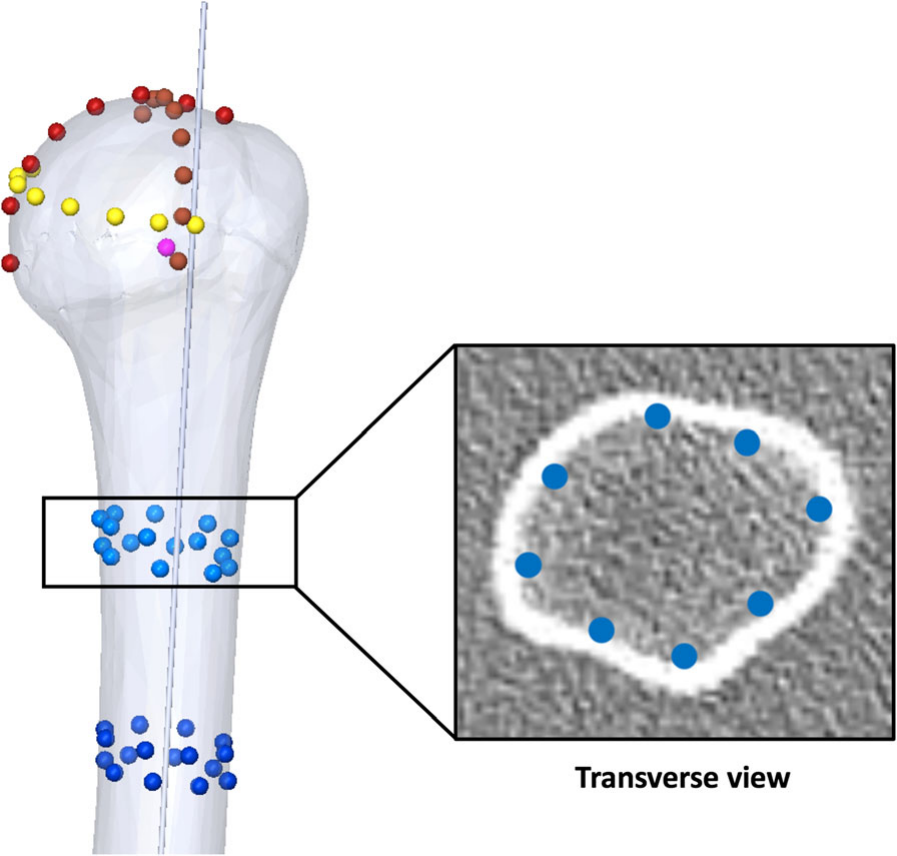
Determination of the humeral head center coordinates by digitizing 3 series of 8 points on the head surface (‘sphere of best fit’ method), and determination of the intra-medullary canal axis and width by digitizing 4 series of 8 points on transverse sections 65, 70, 100 and 105 mm below the head center (‘cylinder of best fit’ method)

**Fig. 2:**
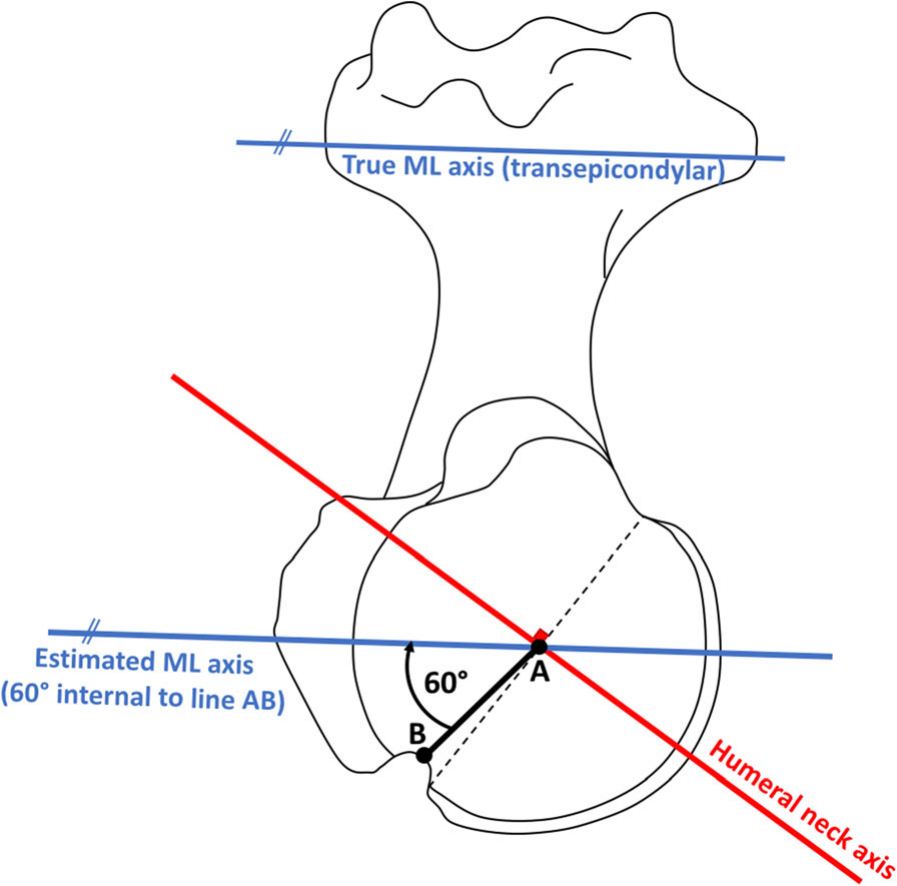
Estimation of the Medio-Lateral (ML) axis based on the centers of the humeral head (**a**) and of the bicipital groove at its proximal portion (**b**)

**Fig. 3:**
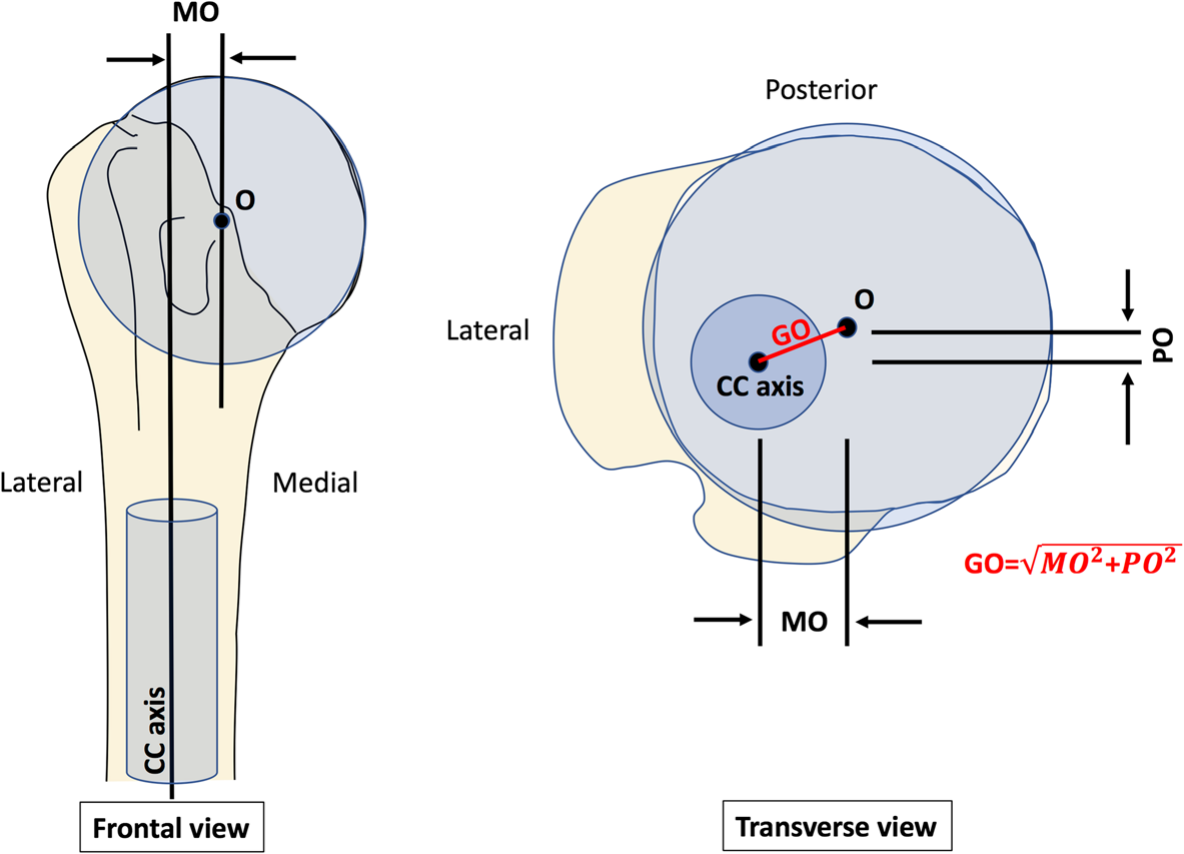
Determination of the medial and posterior components of humeral head offset between the center of the humeral head (O) and the intramedullary canal axis (CC axis). MO, Medial Offset; PO, Posterior Offset; GO, Global Offset

The Digital Imaging and Communication in Medicine (DICOM) files were processed using Osirix (Pixmeo SARL, Bernex, Switzerland) in standard resolution. Four series of 8 points were digitalised on endosteal transverse sections 65, 70, 100 and 105 mm below the head center (Fig. 1), to determine the width of the intramedullary diaphyseal canal, using the ‘cylinder of best fit’ method, and to establish the cranio-caudal (CC) axis. Three series of 8 points were also digitized on the surface of the humeral head (1 series per plane) to determine the humeral head diameter, using the ‘sphere of best fit’ method and to establish the coordinates of the head center. Because the CT scans did not include the elbow joint, it was not possible to establish the mediolateral (ML) axis using the coordinates of the humeral epicondyles. Instead, the coordinates of the centre of the proximal bicipital groove, at the level revealing its full depth, were used to approximate the humeral transepicondylar (TEA), by applying an external rotation of 60° to the line connecting the head center to the proximal bicipital groove (Fig. 2). The approximation was deduced from two recent studies: (i) Johnson et al. (Johnson et al., 2013), who reported the “proximal groove” to be at 60° of internal rotation relative to the humeral TEA; and (ii) Oh et al. (Oh et al., 2017), who found the bicipital groove to be at 60.6° of internal rotation relative to the TEA (“method 2” 30° between bicipital groove and reference line + 30.6° between reference line and TEA). The anterior and posterior boundaries of the anatomic neck were digitized using the limits of the subchondral bone on axial slices, 5–7 mm below the slice where the humeral head has it maximum diameter, and their perpendicular bisector defined the humeral neck axis, which was used to calculate head retroversion with respect to the estimated ML axis. The global head offset was determined by measuring the medial and posterior distances between the humeral head centre and the CC axis in the transverse plane (Fig. 3).

### Statistical analysis

The sample size necessary to test the hypothesis, that there is no correlation (− 0.24 < *r* < 0.24) between native head offset and intramedullary canal width, with alpha = 0.05 and beta = 0.80, was calculated a priori to be a minimum of 71 patients.

The authors selected 13 shoulders at random, for which all parameters were measured by a second observer, to calculate their inter-observer agreement. The intra-class correlation coefficients (ICC) were excellent for intramedullary canal width (ICC = 0.96; CI, 0.60–0.99), head diameter (ICC = 0.97; CI, 0.91–0.99) and global head offset (ICC = 0.85; CI, 0.58–0.95).

Descriptive statistics were used to summarize the data. Shapiro–Wilk tests were used to assess the normality of distributions. For non-Gaussian quantitative data, differences between groups were evaluated using Wilcoxon rank-sum tests (Mann–Whitney U test). For continuous variables, correlations were analysed using Pearsons coefficients. Uni-variable and multivariable linear regressions were performed to determine associations between head offset and five independent variables (gender, age, intramedullary canal width, head diameter, and head retroversion). Both uni- and multi-varaible analyses were deemed necessary to identify potential confounding variables. Statistical analyses were performed using R version 3.3.3 (R Foundation for Statistical Computing, Vienna, Austria). *P*-values < 0.05 were considered statistically significant.

## Results

The average intramedullary canal width was 14.5 ± 2.5 mm (range, 9.4–20.5), and the average head diameter was 44.9 ± 4.4 mm (range, 36.2–56.0), with a retroversion of 24.6° ± 19.2° (range, − 30.7°–59.8°) (Table 2). The mean global head offset was 5.9 ± 1.4 mm (range, 3.4–10.8), with a medial component of 5.1 ± 1.5 mm (range, 2.2–10.7) and a posterior component of 2.2 ± 1.6 mm (range, − 0.9 – 6.4). There were no significant differences in global offset between men and women (5.80 ± 1.45 vs 6.11 ± 1.31; *p* = 0.094).

**Table 2:** Patient characteristics and principal humeral morphometric measurements

	Mean ± SD	Median	Range
Age	59.1 ± 20.5	65.0	(18.0 - 96.0)
Intramedullary canal width	14.5 ± 2.5	14.8	(9.4 - 20.5)
Head diameter	44.9 ± 4.4	45.9	(36.2 - 56.0)
Head retroversion	24.6 ± 19.2	21.8	(−30.7 - 59.8)
Offset	5.9 ± 1.4	6.1	(3.4 - 10.8)
Medial offset	5.1 ± 1.5	5.3	(2.2 - 10.7)
Posterior offset	2.2 ± 1.6	2.0	(−0.9 - 6.4)

**Fig. 4:**
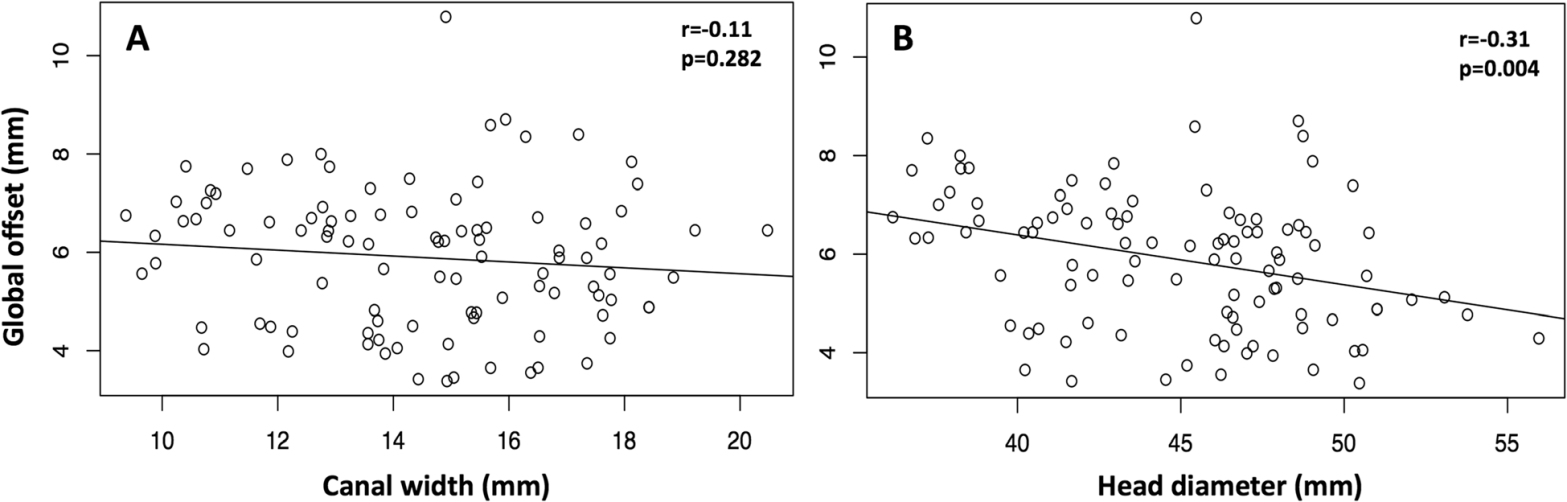
Global humeral head offset for quintiles of intrameduallary canal width (**a**) and humeral head diameter (**b**)

Intramedullary canal width was positively correlated with head diameter (*r* = 0.63, CI = 0.50–0.74; *p* < 0.001). Global head offset was negatively correlated with head diameter (*r* = − 0.31, CI = − 0.48 – -0.13; *p* = 0.002), but not with intramedullary canal width (*r* = − 0.11; CI = − 0.30–0.09; *p* = 0.282), thus confirming the study hypothesis (Fig. 4). Medial head offset was negatively correlated with head diameter (*r* = − 0.28, CI = − 0.45– -0.09; *p* = 0.004), but not with intramedullary canal width (*r* = − 0.13; CI = − 0.32–0.07; *p* = 0.187). Posterior head offset was neither correlated with head diameter (r = − 0.11, CI = − 0.30–0.09; *p* = 0.298), nor with intramedullary canal width (*r* = − 0.05; CI = − 0.24–0.15; *p* = 0.645).

Uni-variable regression revealed that global head offset was significantly associated with head diameter (beta, − 0.10; CI, − 0.16 – -0.04; *p* = 0.001) and patient age (beta, 0.01; CI, 0.00–0.03; *p* = 0.033) but not with intramedullary canal width (beta, − 0.06; CI, − 0.17–0.05; *p* = 0.282) (Table 3). Multivariable regression confirmed that global head offset was independently associated with head diameter (beta, − 0.15; CI, − 0.26 – -0.04; *p* = 0.005), but not with intramedullary canal width (beta, 0.06; CI, − 0.09–0.20; *p* = 0.431), reaffirming the study hypothesis.

**Table 3:** Linear regressions to identify factors associated with global head offset

Variable	Univariable			Multivariable (*n* = 94 shoulders)		
	Regression coefficient	95% C.I.	*p*-value	Regression coefficient	95% C.I.	*p*-value
Male gender	−0.31	(−0.88 – 0.26)	0.289	0.60	(−0.19 – 1.36)	0.130
Age	0.01	(0.00 – 0.03)	0.033	0.01	(−0.01 – 0.02)	0.387
Intramedullary canal width	−0.06	(−0.17 – 0.05)	0.282	0.06	(−0.09 – 0.20)	0.431
Head diameter	−0.10	(−0.16 – -0.04)	0.001	−0.15	(−0.26 – -0.04)	0.005
Head retroversion	0.00	(−0.01 – -0.02)	0.740	0.00	(−0.01 – 0.02)	0.440

## Discussion

The principal finding of our study was that there is no correlation between head offset and intramedullary canal width, thereby confirming our hypothesis. Accurate restoration of anatomy is a prerequisite for the success of TSA (Godeneche et al., 2002; Irlenbusch et al., 2011; Pearl, 2005; Pearl et al., 2009; Wirth et al., 2007). Many anatomic parameters, such as head offset and height, center of rotation, and neck-shaft angle, have been studied to improve the design of anatomic humeral stems (Aroonjarattham et al., 2009; Irlenbusch et al., 2011; Jeong et al., 2009; Kadum et al., 2016; McPherson et al., 1997; Pearl, 2005; Robertson et al., 2000; Roche et al., 2006). While landmark studies reported that humeral head diameter is proportional to intra-medullary canal width (McPherson et al., 1997) and head offset (Pearl & Volk, 1996), this study is the first to investigate the direct relationship between head offset and intramedullary canal width.

It is important to note that, as in numerous published studies, our anatomic parameters were measured on shoulders with normal or healthy bone morphology, whereas the findings are relevant to design prosthetic humeral stems for arthritic shoulders that feature substantial proximal deformations. We chose to study shoulders with normal or healthy bone morphology for two reasons: (i) in arthritic shoulders, subchondral damage to the humeral head often renders calculations of its diameter and centre difficult and inaccurate; and (ii) the goals of TSA are to replace damaged articular surfaces and restore adequate joint architecture, which is often deformed due to congenital or progressive pathologies in arthritic shoulders.

While shoulder arthroplasty was initially intended for elderly patients with low functional expectations, TSA is now performed in younger patients with greater functional demands, which renders reconstruction of native anatomy all the more essential. Modern TSA implants allow for some adjustments of humeral head offset (Irlenbusch et al., 2011; Wirth et al., 2007), but some default designs assume it to be directly proportional to stem diameter, such that larger stems are designed with greater head offsets (Table 1). Our multivariable analysis suggests that this assumption is incorrect, as global head offset was not associated with intramedullary canal width, even when considering the effects of age, gender, and head retroversion.

Our morphologic measurements are within the ranges reported in other published studies (Table 4): 43–51 mm for head diameter (Aroonjarattham et al., 2009; Hertel et al., 2002; Matsumura et al., 2016; Merolla et al., 2008), 16°–31° for head retroversion (Aroonjarattham et al., 2009; Boileau et al., 2008; Harrold & Wigderowitz, 2013; Hertel et al., 2002; Johnson et al., 2013; Matsumura et al., 2016; Oh et al., 2017; Roberts et al., 1991; Robertson et al., 2000), 7–15 mm for intramedullary canal width (Akpinar et al., 2003; McPherson et al., 1997; Murdoch et al., 2002), and 1–4 mm for posterior head offset (Aroonjarattham et al., 2009; Boileau et al., 2008; Hertel et al., 2002; Merolla et al., 2008; Robertson et al., 2000). The medial component of the global head offset was also within the range of 4–6 mm from CT studies (Aroonjarattham et al., 2009; Deladerriere et al., 2012), but below the range of 6–10 mm from X-ray studies (Boileau & Walch, 1997; Hertel et al., 2002; Merolla et al., 2008; Pearl & Volk, 1996; Robertson et al., 2000). This discrepancy is likely due to magnification and rotation in X-ray measurements, which tend to exaggerate the true medial offset. While Pearl et al. (Pearl & Volk, 1996) found the correlation between medial head offset and head diameter to be moderate and positive (*r* = 0.6), we found it to be weak and negative (*r* = − 0.28). These contradictory results might be explained by differences in measurement techniques, as Pearl et al. used cadaver bone x-rays, and referred to the reamed canal for the CC axis.

**Table 4:** Comparison of humeral morphometric measurements reported in the literature

Author	Year	Imagery	Head retroversion		Head diameter		Medial head offset		Posterior head offset		Canal width	
			Mean ± SD (Median)	(min – max)	Mean ± SD (Median)	(min – max)	Mean ± SD (Median)	(min – max)	Mean ± SD (Median)	(min – max)	Mean ± SD (Median)	(min – max)
This study	2018	CT-scan	24.6 ±19.2	(−30.7 – 59.8)	44.9 ±4.4	(36.2 – 56.0)	5.1 ±1.5	(2.2 – 10.7)	2.2 ±1.6	(−0.9 – 6.4)	14.5 ±2.5	(9.4 – 20.5)
Oh et al.	2017	CT-scan	31.4 ±12.1	(10.6 – 56.1)								
Matsumura et al.	2016	CT-scan	32 ±11.0	(3.0 – 62.0)	42.9 ±3.6	(36.6 – 50.7)	6.2 ±1.4	(3.4 – 10.8)	0.9 ±1.1	(−1.9 – 3.6)		
Matsumura et al.	2014	CT-scan	26 ±11.0	(−2.0 – 60.0)								
Harrold et al.	2013	3-D mod	18.5 ±9.0	(2.7 – 37.4)	48.8 ±3.2	(42.7 – 55.1)						
Johnson et al.	2013	CT-scan	21 ±8.0	(0.2 – 34.0)								
Deladerriere et al.	2012	CT-scan					4.1	(1.0 – 10.0)				
Aronjarattham et al.	2009	CT-scan	31 ±2.5	(8.1 – 56.6)	42.7 ±4.2		5.3 ±2.3	(0.1 – 11.0)	3.4 ±2.0	(0.3 – 9.1)		
Merolla et al.	2008	X-rays	(20.0)	(0.0 – 60.0)	(46.0)	(38.0 – 58.0)	(7.0)	(2.0 – 12.0)	(4.0)	(0.0 – 10.0)		
Boileau et al.	2008	CT-scan	16.1 ±13.3	(−17.0 – 44.0)								
Akpinar et al.	2003	CT-scan									7.1 ±1.3	(4.0 – 10.0)
Murdoch et al.	2002	MRI									12.1 ±2.6	(6.0 – 21.0)
Hertel et al.	2002	X-rays			44.5 ±4.0	(36.0 – 57.0)	6 ±1.8	(1.7 – 11.5)	1.4 ±1.4	(−3.0 – 5.3)	11.5 ±2.1	(6.0 – 21.0)
Robertson et al.	2000	3-D mod	19 ±6.0	(9.0 – 31.0)	46.0	(34.0 – 56.0)	7 ±2.0	(4.0 – 12.0)	2 ±2.0	(1.0 – 8.0)		
Boileau et al.	1997	3-D mod	17.9 ±13.7	(−6.7 – 47.5)	46.2 ±5.4	(37.1 – 56.9)	6.9 ±2.0	(2.9 – 10.8)	2.6 ±1.8	(−0.8 – 6.1)		
McPherson et al.	1997	X-rays			47.6 ±4.8		7.6 ±1.5		1.9 ±1.7		15.3 ±2.6	
Pearl et al.	1996	X-rays			50.6 ±4.6	(46.0 – 58.0)	9.7 ±1.7	(6.0 – 12.0)			12 ±1.7	(10.0 – 14.0)
Roberts et al.	1991	3-D mod	(21.4)		(50.3)		(4.7)					

The limitations of this study include: (i) the inability to determine the transepicondylar axis to establish the frontal humeral plane and calculate true head retroversion, (ii) the digitization of endosteal transverse sections at fixed rather than proportional distances below the head center, which was not possible because the total heights of the humerus and of the patient were unknown, (iii) the population studied was Caucasian, and may not be representative of other ethnicities (Aroonjarattham et al., 2009; Matsumura et al., 2016; Zhang et al., 2016), and (iv) the cohort did not comprise any arthritic joints which may have different morphologic characteristics. The main strengths of this study are the use of CT-scans which were demonstrated to be more accurate than X-rays in the assessment of morphology of the proximal humerus (Jia et al., 2016) and a precise measurement method validated by strong inter-observer repeatability.

## Conclusions

The present study revealed that humeral offset is not correlated with intramedullary canal width. These findings are relevant to implant manufacturers and shoulder surgeons, who should be aware of the subtle morphologic features, to enhance humeral stem design and restore native anatomy.

## Abbreviations

CC: Cranio-caudal
CT: Computed tomography
ICC: Intra-class correlation coefficients
ML: MedioLateral
TSA: Total shoulder arthroplasty
